# Safety in home care: A research protocol for studying medication management

**DOI:** 10.1186/1748-5908-5-43

**Published:** 2010-06-04

**Authors:** Patricia B Marck, Ariella Lang, Marilyn Macdonald, Melissa Griffin, Anthony Easty, Serena Corsini-Munt

**Affiliations:** 1 Faculty of Nursing, University of Alberta, Clinical Research Unit, Royal Alexandra Hospital, Alberta Health Services, 6-10 University Terrace, 8303 - 112 Street, Edmonton, AB, T6G 2T4, Canada; 2 VON Canada, 110 Argyle Avenue, Ottawa, ON, K2P 1B4, Canada; 3 Faculty of Nursing, Dalhousie University, 5869 University Avenue, Halifax, NS, B3H 3J5, Canada; 4 Institute for Biomaterials and Biomedical Engineering, University of Toronto; Centre for Global eHealth Innovation, 4 RFE, Toronto General Hospital, 190 Elizabeth Street, Toronto, ON, M5G 2C4, Canada; 5 Institute for Biomaterials and Biomedical Engineering, University of Toronto; Centre for Global eHealth Innovation, 4 RFE, Toronto General Hospital, 190 Elizabeth Street, Toronto, ON, M5G 2C4, Canada; 6 VON Canada, 110 Argyle Avenue, Ottawa, ON, K2P 1B4, Canada

## Abstract

**Background:**

Patient safety is an ongoing global priority, with medication safety considered a prevalent, high-risk area of concern. Yet, we have little understanding of the supports and barriers to safe medication management in the Canadian home care environment. There is a clear need to engage the providers and recipients of care in studying and improving medication safety with collaborative approaches to exploring the nature and safety of medication management in home care.

**Methods:**

A socio-ecological perspective on health and health systems drives our iterative qualitative study on medication safety with elderly home care clients, family members and other informal caregivers, and home care providers. As we purposively sample across four Canadian provinces: Alberta (AB), Ontario (ON), Quebec (QC) and Nova Scotia (NS), we will collect textual and visual data through home-based interviews, participant-led photo walkabouts of the home, and photo elicitation sessions at clients' kitchen tables. Using successive rounds of interpretive description and human factors engineering analyses, we will generate robust descriptions of managing medication at home within each provincial sample and across the four-province group. We will validate our initial interpretations through photo elicitation focus groups with home care providers in each province to develop a refined description of the phenomenon that can inform future decision-making, quality improvement efforts, and research.

**Discussion:**

The application of interpretive and human factors lenses to the visual and textual data is expected to yield findings that advance our understanding of the issues, challenges, and risk-mitigating strategies related to medication safety in home care. The images are powerful knowledge translation tools for sharing what we learn with participants, decision makers, other healthcare audiences, and the public. In addition, participants engage in knowledge exchange throughout the study with the use of participatory data collection methods.

## Background

Patient safety is an ongoing health system priority globally, with medication safety earmarked as a prevalent, high-risk area of concern [1-4]. Maintaining medication safety depends upon the integrity of a complex series of interrelated steps, with failures in assessment, prescription, dispensing, and monitoring potentially leading to adverse events and harm. Human beings and the systems we create are fallible, and at present, we have little understanding of the supports and barriers to safe medication management in the home care environment for clients, family members, or healthcare providers. Compromised medication management can be extremely costly to patients, healthcare professionals, and the healthcare system [4-10]. A recent report concluded that approximately 1.5 million preventable adverse drug events occur per year, resulting in a total cost of $3.5 billion [11]. Similarly, as many as one in five Canadian patients admitted to hospital suffered adverse events within three weeks after being discharge home; 66% of these were medication-related events [12-14]. Moreover, patients recognize problems with the manner in which medications are provided to them. In the 2002 Commonwealth Fund Survey, 11% of patients in Canada reported that they had been given the wrong medication at one time or another [15]. *Patients for patient safety *is one important initiative of the World Alliance for Patient Safety established in 2004 whereby patients and/or their advocates are making themselves knowledgeable about patient safety initiatives, working to inform the public, and participating internationally in the development of safety initiatives [16]. There is a clear need to engage the providers and recipients of care in studying and improving medication safety, and our study contributes to that agenda by adopting a collaborative approach to exploring the nature and safety of medication management for chronically ill seniors receiving home care services.

### Overview of literature: Medication safety for seniors receiving home care

This study addresses a critical gap in our current understanding of medication safety for seniors within the Canadian home care sector. The elderly often manage more than one chronic illness, a complex palate of medications, along with other care needs. The total annual cost of preventable drug-related morbidity (PDRM) in Canada's seniors was estimated to be $11 billion in 2000 [7]. A recent study observed that as many as 1 in 11 seniors in Halifax, Nova Scotia experienced a PDRM over a two-year period [17]. Many of these lapses in safety occur in clients' homes. However, medication safety research to date has focused overwhelmingly on institutional settings that may have little applicability to the home care environment. For instance, most healthcare that takes place in private homes, which are not designed for such activities, is provided by unregulated workers, family, and unpaid caregivers [18]. There is also increasing recognition that traditional methods for classifying drug-related problems including categories such as 'drug without indication,' 'indication without drug,' 'wrong drug,' and 'wrong dose' [19] are not sufficient and practical for home care [20]. For example, social, economic, and community issues are critical when medication-related problems are being assessed. Such issues include: whether the client can afford medication and transportation to fill the prescription; confusion about the purpose, dose, or timing of the medications; and living with an overwhelmed caregiver who has their own health concerns.

Caring for elderly individuals with chronic illnesses in their homes is inherently complex. There has been an increase in the medicalization of private homes, stemming from higher set thresholds for hospitalization, as well as patient acuity at discharge and transfer across care sectors [21]. With the advent of mobile technology (*i.e.*, peritoneal and haemodialysis, long term intravenous catheters and oxygen/inhalation therapy) 'hospital at home' services have seen a huge increase [22]. When providing home care services, it is necessary to consider all factors, including the physical environment, family dynamics, and the cognitive and physical abilities of the client and caregivers. Caregivers, often elderly, are contending with their own health challenges and can lack sleep as they provide care almost 24 hours per day. In contrast, within the institutional scenario there are two or three shifts per day of professionals who provide care. Family members and caregivers often feel a sense of responsibility to attempt to keep the client at home, without fully appreciating that this objective may be unattainable or unrealistic [23,24].

Providers collaborate with clients, families, and their unpaid caregivers to mitigate risks, but the nature of home environments requires clients and caregivers to regularly exercise autonomous decision making about medication use in the context of minimal professional supervision as well as frequently strained or absent home and community supports [18]. In addition, the challenges of documentation and communication, which are heightened at points of transfer across sectors [12-14,25], also increase the potential for inadequate medication reconciliation and its attendant risks. Moreover, the vulnerability of home care staff working predominantly without the proximal supervisory support of colleagues, and the uniqueness of each home setting cannot be overlooked [18,26]. Thus, the care and safety of clients around medication management cannot be attended to without including the family members, unpaid caregivers, and paid providers in the equation [27-29].

### Guiding theoretical framework: a socio-ecological perspective

Our perspective is that the unique nature of private homes and communities as well as the multiple inter-relationships among clients, family, unpaid caregivers, and home care staff constitutes a complex socio-ecological phenomenon in terms of safe medication management. A socio-ecological perspective addresses the influences of social, emotional, functional, and physical contexts of safety pertaining to medication management in home care. Socio-ecological thinking provides a framework for understanding the diverse personal and environmental factors and the interrelationships amongst them, which influence a given health situation [30,31]. Implicit in this perspective on health is the idea that relationships between humans and their environment are reciprocal [30-34]. Many concepts from systems theory are incorporated into this approach in order to elucidate this dynamic relationship [31,35]. Another premise of an ecological perspective is that humans in environments may be described at several levels of aggregation: individual, family, organization, community, and population [34]. Following from this premise is the necessity of both describing the multiple levels of determinants of a problem and identifying opportunities for integrated action across these levels.

### Research questions

The purpose of our research is to understand medication management within the socio-ecological complexity of the Canadian home care environment. Our research questions are:

1. What medication management issues do seniors with chronic illness, their family members, caregivers, and paid providers identify within publicly funded home care programs in four Canadian provinces (Alberta, Ontario, Quebec, and Nova Scotia)?

2. What socio-ecological factors contribute to or reduce the risks of medication management in the home for seniors with chronic illness, their family members, caregivers, and providers in these four Canadian provinces?

3. What strategies do seniors with chronic illness, their family members, caregivers, and paid providers employ to mitigate the risks of medication management at home in the these four Canadian provinces?

4. How do the medication management issues, relevant socio-ecological factors, and mitigation strategies that are identified compare across these four provincial home care programs?

By applying a broad socio-ecological lens to the phenomenon of medication safety in the home, we expect to: illustrate the breadth and depth of risks that seniors, their family members/caregivers, and providers currently encounter in terms of medication management; illuminate the supports and strategies that seniors, their family members/caregivers, and providers presently use to mitigate those risks; and identify potential areas for reducing risks and improving health system supports for medication management in the home.

## Methods/research design

### Setting and sampling strategy

This study takes place concurrently across four Canadian provinces: Alberta (AB), Ontario (ON), Quebec (QC) and Nova Scotia (NS). Purposive and theoretical sampling will be used. Purposive sampling entails deliberatively selecting participants according to the needs of the study [36]. Theoretical sampling will be used once emerging categories related to medication management safety are identified in the initial stages of data analysis in order to direct further sampling to obtain the fullest possible explanation of these categories [36-38]. To ensure maximum variation in this sample, efforts to include seniors of different genders, ages, and home situations will be made. Sampling will continue and be directed by evolving patterns and insights gained from the data. This form of sampling [39] will allow us to verify newly developed patterns, explore relationships among them, and extend our analyses.

In each of the four provinces, we will sample approximately eight households with a chronically ill senior receiving home care services for greater than three months at the time of enrolment in the study. In each household, family member(s) and/or caregiver(s), along with the case manager and paid provider(s) (total approximately three to four participants per household) will be invited to participate in in-depth interviews, providing a potential total of 32 interviews/province or 128 interviews across the four provinces. We will also purposefully sample some clients who experienced a transfer across sectors (*i.e.*, hospitalization and/or ER visit) within the past 90 days of enrolment in the study to obtain insights regarding medication reconciliation and continuity of care as the community dwellers intersect with the acute care sector.

Theoretic sampling based on iterative phases of data collection and analysis poses challenges to predicting the precise number of participants that should be included in the study at the outset of the research. Based on the iterative analysis process in interpretive description (ID) [37,40,41], we acknowledge that we may not have uncovered all patterns relevant to medication safety in home care in each province at 32 interviews. In order to approximate this goal, we will also track outliers in the data. Outliers or rare instances of something will then be considered in the context of identified patterns, and relationships among patterns, to theorize how they may contribute. Selection of arbitrary sample numbers, analyzing the data, and theorizing outliers are deemed in an ID approach to produce findings with clinical relevance, yet do not profess to have uncovered all that is relevant to an issue as does the claim of achieving saturation [40].

### Eligibility criteria

All participants will be able to speak and understand either English or French. Clients will be 65 years of age or older, legally competent, have at least one chronic condition, and be a recipient of home care services, ideally have at least one family member living with them, and have an identified unpaid caregiver. Family and caregiver participants must be at least 18 years of age and legally competent. Paid providers will have worked in home care for a minimum of one year.

### Terminology

It is important to distinguish the terminology used in this proposal. The term 'caregivers' refers to family members or friends, who are in an unpaid role but often are responsible for or charged with caring for the client. Family member(s) are individual(s) identified by the client and/or caregiver as being close to the client through blood, legal, or emotional ties, and who may or may not reside in the same home as the identified client. Providers are the professionals or non-professionals, regulated or unregulated, who are employed by organizations providing home care services (*i.e.*, case managers, nurses, aides, pharmacists, and therapists).

### Methodology and methods

Our qualitative research design incorporates the methodological perspectives of ID and human factors analysis (HFA) into successive, iterative phases of visual and textual data collection and data analysis, using a suite of participatory photographic research methods to illuminate the experiences of clients, caregivers, and home care providers with managing medication in the home from a variety of vantage points.

### Interpretive description

The three central methodological elements of an ID research approach are the objective, the mechanisms, and the product. In this study, the methodological objective is to develop a credible conceptual explanation of the phenomenon of medication management safety at home. The mechanisms used to attain this objective are: identifying and exploring multiple relevant sources of visual and textual data; understanding and comparing across several sources of data, synthesizing the data to capture meanings, constructing theoretical relationships from the data, and explaining the theoretical relationships in ways that are meaningful and applicable in home care. The product of this type of research approach is a theoretical explanation about what is common within the phenomenon under study across participants and their particular contexts [37,41]. The characteristics, patterns, and structure of the phenomena are conceptualized in order to arrive at the explanation.

The theoretical assumptions that underpin ID methodology are that the researcher and participants interact and influence one another in the generation of the data; the experiences of the participants and the researcher are socially constructed and context dependent; and the product or the explanation emerges and is grounded in the data [37,40,41]. These assumptions are congruent with a social ecological framework that seeks to understand the reciprocal relationship between humans and their environment and at various levels of aggregation. ID also calls for the use of multiple methods of data collection, and as such it is particularly suited to this study, which will employ interviews, household photographic walkabouts, and photo narration with clients and caregivers, kitchen table talks using photo elicitation with clients, caregivers and healthcare providers, and photo elicitation focus groups with providers. Generating data through multiple methods enables the researchers to test insights that emerge from the use of one method with the findings that are generated through the application of other methods [37,41].

### Human factors analysis

Human factors (HF) engineering is a scientific discipline that specializes in understanding how humans interact with the world around them. It draws upon applied research in a variety of areas to define the parameters and constraints that influence human behavior in order to design efficient human-centered processes to improve reliability and safety [42]. Healthcare providers vary greatly in their abilities. Lay people, who increasingly represent a growing proportion of caregivers, demonstrate even more variability. The performance and safety profile of the care they provide are often compromised by noise, poor lighting, heat, dirt, improper cleaning products, and moisture. Stress and fatigue, in addition to a lack of preparation and education to manage an array of medications, can also degrade performance over time. A caregiver's performance is directly influenced by the operating characteristics and conditions of the equipment, and the environment and processes involved in medication management. User interfaces that are complex, misleading, or illogical can induce errors in even the most skilled users.

HFA is needed to help health service providers broaden their analyses of medication management safety and to develop more effective and lasting remedies to mitigate the risks to all involved [43], regardless of where the services are provided (*i.e.*, home, community, or institutionalized setting). Throughout the study, focusing on supports and barriers to safe medication management, the HF research team members will examine visual and textual data that:

1. provide a general, preliminary assessment of the home care environment (*e.g.*, low lighting, bright lighting, quiet, loud, hot, cold, many stairs, carpets, hardwood, open concept, narrow hallways, number of stories, presence of outside to inside staircase, ramp, *et al.*).

2. illustrate devices, technology, systems/processes related to care (*e.g.*, a white board to keep track of medications), physical features of the home environment (*e.g.*, stairs), and devices/materials as they are located in the home environment (*e.g.*, where medications are kept and how they are stored).

### Participatory photographic research methods

The collection and analysis of visual images offers a more comprehensive understanding of the complexity of medication management in the home setting. The photographic research methods we will use include:

1. Client and caregiver walkabouts of the home to collect photos that they narrate in relation to the phenomenon.

2. Subsequent kitchen table talks with clients, caregivers, and healthcare providers using interview data, walkabout photos, and walkabout photo narratives to elicit further discussion of safety supports and issues.

3. Photo elicitation focus groups with additional groups of healthcare providers to deepen the analysis of managing medication safety in the home.

Related forms of participatory photo narration where participants collect or lead the collection of images to tell stories about the phenomenon under study have been successfully employed in studies of falls prevention with seniors [44] and in medication safety research conducted in the hospital setting [30]. To extend our understanding of the broader context influencing medication management, we will then review the photos during kitchen-table talks [45] in the client's home with their family, caregivers, and providers in order to elicit further data from them about their experiences of managing medications in the home. In addition, we will conduct focus groups with regulated providers (*i.e.*, nurses, pharmacists, physicians, physio-therapists, *et al.*) and non-regulated healthcare aides and homemakers, using select photos and quotes from the visual and textual data to elicit discussion about the challenges surrounding medication management in home care and will validate researcher interpretations of the data gathered during initial in-depth interviews, photo walkabout narratives, and kitchen-table talks.

### Participant recruitment

The research assistant (RA) will work with the case managers from the participating organizations (*i.e.*, Alberta Health Services, VON Canada in Ontario and Nova Scotia, and the Centre for Social and Health Services Cavendish in Quebec) to help identify and recruit participants. Case managers will briefly describe the study to the client and family/caregivers, in person or by phone, and ask permission to release their name to the researcher. During the initial contact with the case manager, potential participants will be reassured that accepting to release their names to the RA will not obligate them to participate in the study. Those who agree will be contacted by the RA who will explain the study in greater detail, answer any relevant questions, and establish verbal consent for those who are expected to participate. All participants will sign formal consent forms prior to each interview.

With the exception of the client, who will be interviewed in his or her home, other interviewees will be interviewed at a time and location convenient to them. The researchers recognize that there are topics that may be sensitive, confidential, and/or private in nature and that participants may prefer not to share information in the presence of other participants (especially related to: potential abuse, fatigue, frustration, *et al.*). Should the family member(s) and/or caregiver(s) prefer to be interviewed in the client's home the interviewer will make efforts to help select a physical space that will ensure maximum possible privacy for the interviewees. Potential provider participants will receive a summary of the project and whom they should contact regarding their participation in the interview and/or focus groups.

### Phases of iterative data collection and analysis

At the onset and throughout the research, the provincial study leads in each province will engage in periodic immersion in the field to supervise data collection and to gain familiarity with the emerging issues. Consistent with the theoretical underpinnings of ID, three successive phases of data collection and analysis will be directed in a concurrent, iterative fashion [37,41] as follows:

### Phase A - component one

Audio-taped, in-depth, semi-structured interviews will be conducted in an approximate total of 32 households (eight households across four provinces) with three to four participants per household (one client, one or two family/caregiver(s), one case manager, and one or two provider(s). This interview component will be conducted by a study lead and a trained research assistant with the support of two tools:

1. Semi-structured interview guide designed to facilitate dialogue with the participants about their respective views of medication management in the context of home care.

2. Checklist of HF probes that incorporates a preliminary HF assessment of the client and their home situation from each participant's perspective--*e.g.*, is the client hard of hearing? Does the client have vision loss? Does the client use a wheelchair? How are medications stored? This will provide some context related to everyday life in that household. If relevant HF topics do not emerge during the initial stages of the interview, the checklist will help the researchers include these topics in the discussion.

### Phase A - component two

Audio-taped photo narrated walkabouts will be conducted in the homes of the clients. Adapting from photo narration methods and field protocols used in previous safety research in the community [44] and acute care [30], we will conduct audio-taped client and family caregiver-led walkabouts of client participants' homes during the same initial home visit for interviews. In a select number of Ontario households, a human factors engineer (HFE) co-investigator will accompany the study lead and RA to assess the home environment for medication management from a HF perspective. During these walkabouts, participants will verbally narrate and visually guide the study lead and RA through their day-to-day experience of managing medication safety. The research assistant and HFE, when present, will participate in observational analyses during these walkabouts. The RA will also collect still digital photographs of areas of the home that participants identify as significant to managing medication safety. Semi-structured questions and a list of prompts will be used to seek and audio-record the client's and/or caregiver's perspectives on the emotional, social, functional, economic, physical, and environmental challenges that they encounter as they try to manage medication safely as well as the coping strategies that they use to identify and mitigate risks. Permission will be sought to take additional photographs in the event the RA or HFE identify noteworthy areas.

The interview data collected will be assigned file numbers, backed up, professionally transcribed, and then uploaded into password protected MS Word files for hand analysis purposes as well as into text files in NVivo8 [46]. As interview transcripts and field notes become available, members of the research team will first independently read and reflexively annotate the transcripts/field notes several times and then provide their respective preliminary coding of the transcripts they have reviewed to the RAs to enter into NVivo8. The strategy of using two or more people to independently code and analyze the data and using other team members to code and analyze a sub-set of data is an example of a successful analysis strategy employed in previous studies [47]. Using the data management capabilities of NVivo8, team members will then subsequently convene to identify recurring, converging, and opposing patterns, relationships among patterns, illustrative examples, linkages to theory, practice, and policy, as well as issues for further group deliberation.

The RAs will upload, back up, and store the photographic data (jpeg files) and the transcribed photo narration data and field notes that we collect in component two walkabouts into NVivo8 in accordance with previously used research procedures [44]. We will then repeat a version of the phase A - component one process where team members first independently and concurrently analyze and code the visual and written texts and then collectively re-analyze the visual and written data in NVivo8. This part of the analysis will include evaluating photos of specific medication management strategies in the home environments using principles of socio-ecological theory and HF (such as usability heuristics) to objectively identify latent safety issues [48]. Examples of heuristics include consistency, visibility, efficiency, flexibility, and error prevention.

To identify areas of similarity and difference among clients, family members, caregivers, providers, as well as across home care service delivery models in the four provinces, the team will then conduct coding comparisons to identify coding discrepancies. Coding comparisons enrich the analysis by including aggregate data, multiple interpretations, and cross-referencing [49]. Discrepancies in coding will lead to the development of new coding categories and define areas for further exploration. As the volume of data increases, is reviewed, re-reviewed, and then coded, categories will be revised and refined in an iterative process among the research team members. As exemplars from coded categories are retrieved and compared within and across the written and visual text for the initial walkabouts, we will incorporate questions about the categories that emerge into subsequent interviews with remaining walkabout participants in order to expand findings and check descriptive and interpretive credibility [37,50]. We will also track outliers in the data set.

Following the piloting of data collection and data analysis for components one and two with two households in Ontario by one study lead, an RA, and a HFE, we will review and modify the data collection tools for components one and two as indicated. Additional initial home visits (components one and two) and subsequent home kitchen table talks (component three) will then be conducted with households as participants are recruited in each of the four provinces, using an iterative approach to data analysis within each case and across cases as we proceed in the four provinces. The provincial research teams will meet every two to three weeks throughout data collection and analysis to allow for ongoing iterative comparison of emerging patterns and relationships in their respective data sets, and to theorize about respective outliers.

### Phase B - component three

Based on the data analyses that we generate in phase A, we will design and conduct three audio-recorded kitchen table talks per province with two to four participants per household. During these kitchen table talks, the RA will review selected photos of the client's home with the client and at least one to two other caregiver(s) and/or provider(s) in order to observe them and their inter-relational dynamics [45] in the setting where services are delivered, and to elicit and stimulate group discussion and further narrative accounts of medication safety and concerns regarding medication management from the various discussants' perspectives.

We will then repeat a third round of independent analyses of the visual and textual data followed by a collective team analysis in NVivo8 to arrive at our preliminary findings about the data from the first two phases of the research. When the pan-Canadian team can demonstrate a robust, credible interpretation of the data from phases A and B across all four provinces, phase C will be initiated in all of the provinces to conduct the fourth component of data collection, photo elicitation focus groups, to triangulate with the household data and strengthen the credibility of the analytic process.

### Phase C - component four

To assess the confirmability of the findings from the first two phases of iterative data collection and analyses, the study leads will conduct two audio-taped focus groups with home care providers in each province. One focus group will be conducted with six to eight regulated providers and one with six to eight unregulated providers. A total of eight focus groups will be conducted across all provinces. Using selected photographic data and our evolving interpretive and HF analyses, we will elicit providers' perceptions of risks and supports related to medication management safety in home care. Each focus group will last a maximum of two hours. These providers will not be asked to directly recount their experiences with one specific case, but rather asked to provide information to enrich the findings to date from the first three components by describing and discussing their experiences in general around medication management in home care. Field notes will be recorded after each focus group.

Initially, phase C focus group transcripts with accompanying field notes will be independently analyzed by the study leads for each province. Study leads and HF team members will then jointly analyze the transcripts to derive a multi-dimensional narrative of medication management in the home from the perspectives of the client, caregiver, and/or family member, and paid provider. It is also important to note that the focus groups will also serve an important knowledge translation opportunity in this study, because the home care providers are acting as participants and stakeholders in this research, they also become a vehicle for knowledge dissemination.

Throughout all phases of data collection, the RAs, HFE, and other investigators will enhance the visual and verbal data that is collected by recording field notes using a standardized template. The study leads and research team members will also assign planned periods of data analysis between each step during the data collection phase to ensure adequate integration and synthesis of data into the next step of the research, with the study leads maintaining and sharing ongoing notes about the emerging analyses and syntheses with the full research team for feedback. Interim analyses will be shared with the whole research team for feedback and critique to enhance the rigor of emerging interpretations of the data. This stepwise analysis will continually inform research decisions about sampling and guide supplemental modifications to protocols at each step. For example, congruent with the iterative process of ID, as data collection and preliminary analysis progress, we will modify successive protocols for the interviews, walkabouts, kitchen table talks, and focus groups in light of emerging themes in order to seek further insights, test preliminary findings, and look for commonalities and/or differences among participants.

### Scientific integrity

The research has received ethical review and approval from the participating universities and service providers (McGill University, University of Ottawa, Dalhousie University, University of Alberta, VON Canada, CSSS Cavendish, and CSSS Bordeaux-Cartier-Ville St-Laurent). Written informed consent will be obtained from all participants and confidentiality will be assured and preserved in all cases. The study has relied on strong communication and interdisciplinary collaboration from conception to realization. In addition to bi-weekly meetings held between the provincial study leads (AL, MM, and PM), the research team has utilized several different forms of communication to navigate operational issues with all collaborators. Through the co-principal investigator at the University of Alberta, the team has access to a web-based login system, E-Class, in which drafts of current papers, ethics proposals, and protocols can be easily shared between all members of team. Additionally, through E-Class, the research team can meet virtually and use screen sharing to collaborate on documents in real-time. All collaborators are kept up-to-date with quarterly e-newsletters.

The rigor of the interpretations that are developed in this study is strengthened by input at varying stages from research team members with expertise in one or more of nursing, medicine, pharmacy, HF engineering, healthcare ethics, law, patient advocacy, healthcare administration, home care practice, and qualitative and visual methods. Attention to rigor will be reflected by openly accounting for the impact of bias (personal, discipline/profession-based, *et al.*) on study results, recording clear documentation about the research-gathering contexts and procedures, and creating a logically auditable path from data collection to conclusion phases [49,51]. The research team will also optimize the trustworthiness of the study findings by means of the following measures; credibility, transferability, dependability, and confirmability [51,52].

Credibility means that the processes used to carry out the inquiry are made explicit and the findings are approved by the constructors. In this study, we will achieve credibility by immersing ourselves in the data to come up with tentative explanatory notions that are then subjected to peer scrutiny and returned to the co-constructors of the explanations through the kitchen table talks, providing a test of the researcher's interpretations. A further test of credibility has been incorporated into this study through consultation at various stages of data analysis with an advisory panel of experts in the delivery of home care services who are also part of the Canadian Patient Safety Institute's (CPSI) Core Team for Safety in Home Care.

Transferability addresses the question as to whether or not the study findings are applicable in settings other than those studied. In order to enhance transferability, we have thoroughly described our methods and will pay particular attention in the writing of our findings to describe in detail the conceptual path we follow to arrive at our explanations. If this process is adequately described, those reading the findings will be able to make decisions about the transferability of the findings of this study to other settings.

Dependability is assured by demonstrating that a second researcher following the same methods and processes as the first will uncover similar findings. This study is ideally structured to meet this criterion with researchers collecting data independently in four provinces. Each researcher will collect and analyze data for their respective province with ongoing access to the expertise of the HFE before any cross-province comparisons are made. This format assures that we will meet the criterion of dependability.

Confirmability is established when there is agreement among the researchers that the knowledge generated is meaningful to clients and care providers as well as being applicable in the home care setting. Confirmability will be arrived at by assuring credibility, transferability, and dependability.

## Discussion

This project is original, timely, and pertinent, given the recent national initiative highlighting the urgency to advance our understanding of the issues, challenges, and risk-mitigating strategies related to safety in home care, particularly around medication management. It highlights the need to view safety through a new lens in order to attend to the complexity, multidimensionality, and distinctness of safety in home care as compared to patient safety in institutions while addressing the importance of continuity across the continuum of care. Consideration of HF principles contributes unique and diverse perspectives to the research findings, and the use of ID, with a variety of methods of data collection procedures, brings a fitting and rich approach to the research question. Furthermore, strong and strategic links with decision- and policy-makers as co-investigator and collaborators from the project's inception, including provincial health ministries, national health information, patient safety, and accreditation bodies, and provincial and federal funding partners optimizes feasibility and knowledge translation capacities. An additional strength stems from engaging participants in knowledge translation throughout the study with the use of participatory data collection methods. The shared interaction and influence between the researchers and participants will generate data and produce findings that are meaningful and useful to the participants and to all stakeholders.

## Competing interests

PM holds a joint appointment with one of the partner agencies, Alberta Health Services, and AL is a nurse scientist with one of the partner agencies, the Victorian Order of Nurses. All three study leads (PM, AL, and MM) participate regularly in a variety of safety initiatives sponsored by one of the partner agencies, the Canadian Patient Safety Institute.

## Authors' contributions

AL, MM, and PM conceptualized and co-designed this study in consultation with AE and other team members, prepared the proposal, and obtained funding. AL holds the study funds at the Victorian Order of Nurses, oversees the financial management of the project, and is study lead for the Ontario and Quebec data collection and analysis. MM is study lead for the Nova Scotia data collection and analysis, and PM is study lead for the Alberta data collection and analysis. The three co-principals will co-lead the cross-province data synthesis. PM and MM co-led the design of the methods portion of the proposal. PM led the preparation of the study protocol and this manuscript. All co-authors are taking part in data collection, data analysis, report-writing and dissemination activities. All co-authors read and approved the final version of this manuscript.
